# PDE4A knockdown rescues alcohol-induced cognitive impairment and synaptic dysfunction via the cAMP/PKA/CREB pathway

**DOI:** 10.1093/ijnp/pyag031

**Published:** 2026-07-11

**Authors:** Huan Tu, Rongzhen Sun, Shengyao Ma, Chunxu Li, Mingti Lv, Mei Han, Han-Ting Zhang

**Affiliations:** Department of Pharmacology, Qingdao University School of Pharmacy, Qingdao 266073, China; Qingdao Hospital, University of Health and Rehabilitation Sciences (Qingdao Municipal Hospital), Qingdao 266071, China; Department of Pharmacology, Qingdao University School of Pharmacy, Qingdao 266073, China; Department of Pharmacology, Qingdao University School of Pharmacy, Qingdao 266073, China; Department of Pharmacology, Qingdao University School of Pharmacy, Qingdao 266073, China; Department of Pharmacology, Qingdao University School of Pharmacy, Qingdao 266073, China; Shandong Key Laboratory of Pathogenesis and Prevention of Brain Diseases, Qingdao 266073, China; School of Pharmacy, Jiangxi Medical College, Nanchang University, Nanchang 330031, China

**Keywords:** alcoholic dementia (AlD), phosphodiesterase-4A (PDE4A), 3xTg-AD mice, cognitive deficit, synaptic damage

## Abstract

**Background:**

Alcoholic dementia (AlD) is a severe neurological disorder with no effective treatment. Development of pan-phosphodiesterase-4 (PDE4) inhibitors for clinical treatment of neurological conditions has been hampered by emetic side effects primarily mediated by PDE4D, one of the four subtypes of PDE4. Although PDE4A was shown to be upregulated by chronic alcohol exposure, its therapeutic relevance to AlD remains unclear.

**Methods:**

A stable mouse model of AlD was established in 3-month-old 3xTg-AD mice using a free-choice two-bottle protocol (25% alcohol) for 19 weeks. Mice received either rolipram injections for 3 weeks or PDE4A knockdown for 6 weeks. Cognitive function was assessed using the Morris water maze, novel object recognition, and Y-maze tests. Hippocampal neuropathology—including Aβ deposition, synaptic integrity, Tau phosphorylation (at Ser214/Ser404), and neuronal apoptosis—was evaluated by immunofluorescence or immunohistochemistry. Changes in key proteins of the cAMP/PKA/CREB signaling pathway were analyzed by Western blotting.

**Results:**

This study demonstrated that selectively targeting PDE4A rescued cognitive and neuropathological deficits in a validated mouse model of AlD. Behavioral tests showed that PDE4A knockdown significantly improved spatial memory and cognition in the Morris water maze, novel object recognition, and the Y maze in the AlD mice. Pathological analysis revealed that PDE4A knockdown reduced amyloid-beta (Aβ) deposition and phosphorylated Tau levels. Furthermore, it restored synaptic integrity, as evidenced by the upregulation of PSD95 and Synaptophysin, enhanced dendritic complexity, and increased spine density, while also exerting neuroprotective effects by suppressing neuronal apoptosis in AlD mice. Mechanistically, the therapeutic benefits of PDE4A knockdown were mediated through the reactivation of the critically impaired cAMP/PKA/CREB signaling pathway, leading to elevated cAMP levels, increased PKA activity, and enhanced phosphorylation of CREB.

**Conclusion:**

Our findings established PDE4A as one of the key AlD pathological drivers and identified its selective inhibition as a novel and promising therapeutic strategy for AlD, offering a viable approach to circumvent the adverse effects associated with broad-spectrum PDE4 inhibition.

## Graphical Abstract

**Graphical Abstract:**
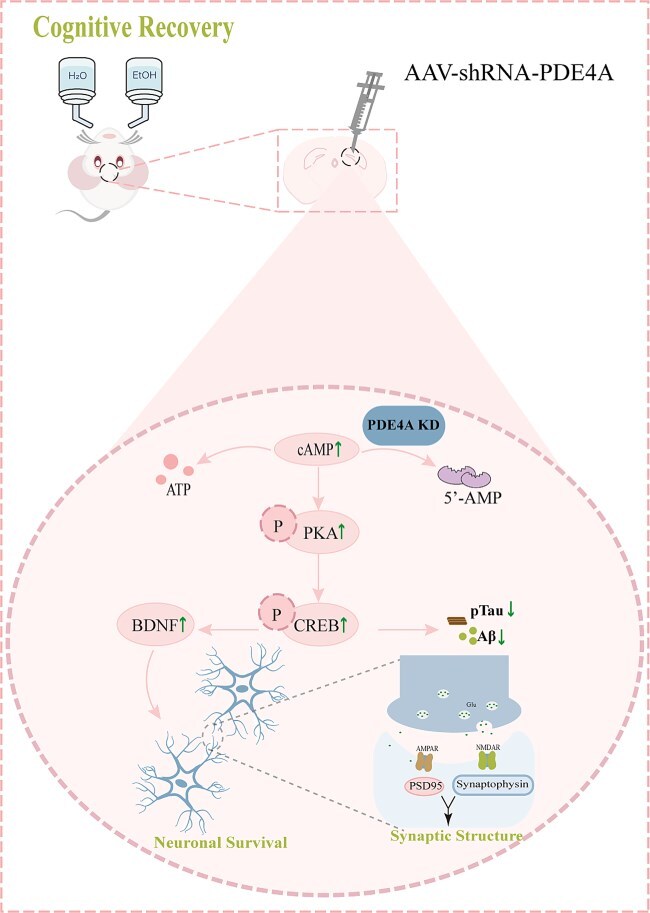

## Teaser

**Highlights**
 

- PDE4A knockdown improved cognitive impairment in AlD mice.
- PDE4A knockdown attenuated Aβ pathology and Tau hyperphosphorylation.
- PDE4A knockdown activated the cAMP/PKA/CREB signaling pathway, alleviating synaptic and neuronal damage in AlD mice.

**Significant outcomes**

Alcoholic dementia (AlD) is a severe neurological disorder for which there is no effective treatment. Although phosphodiesterase 4 (PDE4) inhibitors have shown neuroprotective potential in preclinical studies, their clinical translation is limited by emetic side effects, primarily mediated by the PDE4D subtype. This underscores the need for subtype-selective targeting. We previously found that chronic alcohol exposure specifically upregulates the PDE4A isoform, positioning PDE4A as a compelling and novel therapeutic target for AlD. In AlD mice, PDE4A knockdown significantly rescued cognitive function, reduced Aβ deposition and Tau phosphorylation (Ser214/Ser404), and restored synaptic integrity. It also promoted an anti-apoptotic shift, resulting in reduced neuronal loss. Mechanistically, PDE4A knockdown restored the impaired cAMP/PKA/CREB signaling pathway. Our findings establish PDE4A as a key driver in AlD pathology and support its selective inhibition as a promising therapeutic strategy.

**Limitations**

AlD mouse models were established using 3xTg-AD mice. Owing to the presence of human APP/PS1 and Tau mutations, these mice exhibit spontaneous, age-dependent progression of Aβ deposition, and Tau hyperphosphorylation. This limits generalizability to broader AlD pathology and complicates the distinction between direct alcohol neurotoxicity and transgene-driven pathology. Future studies using non-transgenic models (eg, C57BL/6J) are needed. Although PDE4A knockdown reduced alcohol intake, it remains difficult to fully dissociate direct neuroprotective effects from secondary effects due to reduced ethanol exposure. Nevertheless, the robust beneficial effects on synapses, Aβ, and Tau were observed. Passive alcohol administration models (eg, vapor or gavage) are warranted. In addition, sex differences influence alcohol-induced neurotoxicity (female higher intake, male earlier cognitive impairment), but sex was not included as a statistical variable; future studies should address sex-dependent effects. Finally, no direct pathway interruption experiments (eg, PKA inhibitor) were performed. However, the observed activation of the cAMP/PKA/CREB pathway, together with the neuroprotective outcomes, supports a mechanistic involvement of this signaling cascade in the effects of PDE4A knockdown. Direct causal evidence requires further validation.

## INTRODUCTION

Chronic excessive alcohol consumption represents a major global public health challenge. According to the World Health Organization (WHO), by 2023, alcohol use is responsible for ~3 million deaths annually, accounting for 5.3% of all global mortality.^1^^,^^2^ Among the most severe neurological consequences of long-term alcohol abuse is alcoholic dementia (AlD), a progressive cognitive dysfunction syndrome characterized by memory impairment, personality changes, and intellectual decline that comprises roughly 10% of all dementias.^3^^,^^4^ Compelling epidemiological evidence further establishes a causal link between alcohol dependence and an accelerated onset of Alzheimer’s disease (AD), with the risk increasing proportionally to the duration of alcohol abuse.^5–7^ Despite its clinical significance, the precise pathophysiological mechanisms underlying AlD remain incompletely elucidated, hampering the development of effective therapies. Current understanding points to alcohol-induced neuronal damage and the loss of synaptic plasticity as central contributors to its pathogenesis.^8^^,^^9^ In view of this, it is particularly important to systematically study the pathogenesis of AlD and explore potential therapeutic targets.

Neurons and their synaptic connections are fundamental substrates of learning and memory, forming dynamic networks that encode cognitive experiences. The ability of these synapses to strengthen or weaken over time—known as synaptic plasticity—is widely regarded as the primary cellular mechanism underlying memory formation. Existing studies have confirmed that ethanol and its metabolites (especially acetaldehyde) directly impair neuronal structural integrity and suppress synaptic plasticity.^10^^,^^11^ Neuroimaging studies have consistently revealed hippocampal atrophy and a characteristic loss of synaptic density in chronic alcoholics, reflecting the degeneration of the very circuits that support cognitive function.^12^^,^^13^ Furthermore, alcohol intake accelerates the pathological accumulation of amyloid-beta (Aβ) and hyperphosphorylated Tau in the hippocampus.^14^^,^^15^ The soluble oligomers of these proteins not only are neurotoxic but also directly disrupt synaptic signaling, providing a potential mechanistic bridge between alcohol abuse and Alzheimer’s-like pathology.^16^^,^^17^

Within the complex landscape of intracellular signaling, the cyclic adenosine monophosphate (cAMP) signaling pathway plays a pivotal role in neuronal survival, plasticity, and cognitive function.^18^ Phosphodiesterase (PDE) is a key hydrolase that selectively degrades cyclic nucleotides (cAMP/cGMP) and affects cell signal transduction by precisely regulating the levels of the second messengers. The currently known 11 PDE families (PDE1-11) together constitute a complex cyclic nucleotide regulatory network through their tissue-specific distribution and functional differences. Among them, PDE4, as the most important subtype in the central nervous system, dominates 70%-80% of cAMP metabolism in neurons and is the core regulatory factor for maintaining cAMP homeostasis in the brain.^19^ Preclinical studies have demonstrated that broad-spectrum PDE4 inhibitors suppress drinking behavior and confer cognitive benefits.^20^^,^^21^ However, their clinical translation has been fundamentally obstructed by severe, dose-limiting side effects, most notably nausea and vomiting, which are primarily mediated by the PDE4D subtype expressed in the brainstem’s vomiting center.^22^

This necessity for subtype selectivity has directed attention to the distinct roles of PDE4 family members (PDE4A-D). While PDE4A, PDE4B, and PDE4D are all expressed in the central nervous system, they exhibit unique distribution patterns and functional specificity. PDE4B is predominantly localized to microglia and astrocytes, modulating neuroinflammatory pathways such as NF-κB.^23^^,^^24^ In contrast, both PDE4A and PDE4D are highly enriched in neurons of the hippocampus and cerebral cortex,^25^ where they critically regulate the cAMP-protein kinase A-cAMP response element-binding protein (PKA-CREB) signaling cascade, a pathway essential for synaptic plasticity, long-term potentiation, and memory consolidation. The functional abnormalities of PDE4A and PDE4D are closely related to the pathological processes of various neuropsychiatric diseases such as depression, anxiety,^26^ and AD.^27^^,^^28^ Crucially, PDE4D is abundantly expressed in the area postrema nucleus of the solitary tract, explaining the emetic response to its inhibition.^29^ PDE4A, however, shows minimal presence in these regions, offering a compelling therapeutic advantage by potentially circumventing the debilitating side effects that have plagued previous PDE4 inhibitor development. Supporting this premise, our preliminary investigations identified a significant upregulation of PDE4A expression and activity in the mice model of AlD, positioning it as a promising target.^30^

Based on this rationale, the present study aimed to rigorously evaluate the therapeutic potential of selectively targeting PDE4A in AlD. We employed a well-established two-bottle choice chronic drinking paradigm to induce an AlD-like condition in mice.^5^ Using a genetic knockdown approach, we systematically assessed the impact of PDE4A suppression on voluntary alcohol consumption, cognitive performance in learning and memory tasks, and key neuropathological hallmarks. Furthermore, through a combination of immunohistochemistry and western blotting analyses, we investigated the effects on neuronal function, synaptic ultrastructure, and the activity of the downstream cAMP-PKA-CREB signaling pathway to elucidate the molecular mechanisms underpinning the observed phenotypic outcomes. Our data supported PDE4A as a novel and highly selective target for mitigating alcohol-induced cognitive decline, offering a viable strategy to overcome the limitations of pan-PDE4 inhibition.

## METHODS

### Animals

Triple transgenic mice model (3xTg-AD) carrying three mutant genes (PS1_M146V_, APP_swe_, and Tau_P301L_) associated with human familial AD and was purchased from the Jackson Laboratory in the United States. The transgenic mice populations were maintained by continuous inbreeding, and all experimental mice were homozygous for the above three mutant genes.^31^ Control group used wild-type (WT) mice with the same genetic background (C57BL6/129SvJ mixed background) and matched age. Experimental design adopted the strict random grouping principle, and 3-month-old mice were divided into 7 experimental groups (*n* = 10 in each group, half male and half female): WT mice given normal drinking water (WT + W), WT mice given alcohol intervention (WT + A), transgenic mice given normal drinking water (3xTg-AD + W), transgenic mice given alcohol intervention (3xTg-AD + A), transgenic mice alcohol intervention + adeno-associated virus control (3xTg-AD + A + NC), transgenic mice alcohol intervention + PDE4A knockdown adeno-associated virus (3xTg-AD + A + 4A-KD) and transgenic mice alcohol intervention + rolipram drug treatment (3xTg-AD + A + R). All experimental animals were housed in a standard SPF environment, using an independently ventilated cage system (IVC), maintaining a 12/12-h light–dark cycle (light period 08:00-20:00), and free access to food and water. Animal experimental protocol was reviewed and approved by the Experimental Animal Ethics Committee of Qingdao University and was carried out in strict accordance with the requirements of the International Guide for the Care and Use of Laboratory Animals.

### Two bottles choice test

In this study, 3-month-old 3xTg-AD transgenic mice and WT control mice were used to establish an AlD model using a 19-week two-bottle choice paradigm. These mice were exposed to two 100 mL flasks containing either a sugar-containing alcohol solution (25% ethanol w/v + 0.1% saccharin w/v) or pure water for 24 h a day for 19 weeks until they are euthanized; the placement of the bottles (left/right) was alternating every 24 h to eliminate position preference, and fluid consumption was recorded every 1 week.

### Stereotaxic brain injection

AAV2/9-m-PDE4A/NC-shRNA-EGFP was purchased from HANBIO. Mice were anesthetized with isoflurane in a cage and placed in a stereotaxic frame (RWD Life Science, China). Anesthesia was maintained using isoflurane (1%) inhalation. Fur was removed from the top of the skull with scissors, and the area was disinfected with iodine. A skin incision was then made, and small holes were drilled at X (±1.15 mm from bregma) and Y (−1.7 mm from bregma). AAV-PDE4A-shRNA and isotype control were delivered into the right and left hippocampus at a Z depth of 1.5 mm at 0.10 μL/min, respectively. After injection, the syringe was left in place for 10 min and then slowly removed. The wound was sutured, and the mice were returned to the cage and observed for 1 h. The mice had 6 weeks to recover after surgery.

### Drug treatment

Rolipram was purchased from TargetMol. The drug solution was prepared using physiological saline containing 1% dimethyl sulfoxide (DMSO) as the solvent. 3xTg-AD + A + R mice began drug treatment after completing a two-bottle choice experiment for 16 weeks. The intraperitoneal injection was performed at 8 a.m. every day, and the administration volume was uniformly 1 mg/kg. The other groups were given an equal volume of physiological saline solution.

### Behavioral testing

Behavioral tests were initiated 24 h after the 10th intraperitoneal injection of rolipram. The experimental sequence was the Morris water maze (MWM) test, novel object recognition (NOR) test, and Y-maze test. To eliminate the interference of the acute sedative effect of the drug, all behavioral tests were started 1 h after drug administration. Between each test, the experimental animals were returned to the original breeding environment for adaptive rest.

#### Morris water maze

This was used to evaluate the spatial learning and memory ability of mice.^32^ The experimental setup consisted of a circular pool 120 cm in diameter and 50 cm high (water depth 30 cm, water temperature 21-23°C), filled with an opaque liquid, placed in a laboratory with spatial cues. The experimental space was divided into four quadrants, with a hidden platform 8 cm in diameter fixed in the center of the third quadrant, its surface 1 cm below the water surface. The experiment was divided into two phases. The first phase was the training phase, lasting 5 consecutive days. Mice were tested four times a day, entering the water from the first, second, third, and fourth quadrants, respectively. Each trial required the mice to swim for 60 s to find the hidden platform. If the mice found the platform within 60 s, they were allowed to remain on it for 30 s. If they failed to reach the platform within the time limit, they were gently guided onto it and remained there for 30 s. The second phase was the testing phase. In this phase, the platform was removed, and the test was performed only once. The Topscan Software Package (Clever Sys, USA) was used to record the number of times mice crossed the platform, their swimming speed, and the exploration time in the target quadrant within 60 s for statistical analysis.

#### Novel object recognition

The NOR experimental system consisted of a 40 × 40 × 40 cm behavior box and a video acquisition system. The experiment employed a standard two-stage paradigm. The first stage was the training phase. Two identical black cylinders, A1 and A2 (5 × 7 cm), were symmetrically placed inside the experiment box. Mice were placed in the open box and allowed to explore freely for 5 min. After 5 min, the mice were removed, and the experiment box and objects were cleaned with 75% ethanol to avoid odor interference. The second stage was the testing phase, conducted 1 h after the training phase. One of the black cylinders in the experiment box was replaced with a yellow cube B (5 × 5 × 7 cm). The mice were allowed to explore freely for 5 min, and their movements were tracked and recorded by a camera. Finally, the video was analyzed using VisuTrack software. The discrimination index (DI) = (new object exploration time / total exploration time) × 100%.

#### Y-maze test

The Y-maze experiment consists of three equal-length acrylic arms (each arm’s length × width × height: 32 × 8 × 15 cm), arranged at a 120° angle. The experimental setup is placed in a laboratory with constant temperature (21-23°C) and soft, uniform lighting. Mice were initially placed in the starting arm A. Of the other two arms, one is closed, called the novelty arm C, and the other is the familiar arm B. The Y-maze experiment is divided into two phases. The first phase is the training phase, in which mice were placed at the end of the starting arm and allowed to freely explore the starting arm, the familiar arm, and the central area for 5 min. After 2 min, the testing phase begins, and the novel arm C is opened. The mice were then placed back at the end of the starting arm in the same manner and allowed to freely explore all three arms for 5 min. During this period, the mice’s movement trajectory is tracked and recorded. After each trial, each arm is wiped with a 75% alcohol solution to remove olfactory cues. The time each mouse spends in each arm of the Y-maze is calculated using Visu Track software.

### Tissue preparation

After behavioral testing, mice were deeply anesthetized with isoflurane, and their whole brains were removed via cardiac perfusion with cold 0.9% saline and paraformaldehyde fixation. Hippocampal tissue for immunofluorescence staining was first fixed overnight in 4% paraformaldehyde and then dehydrated in sucrose solution for at least 48 h. The brain tissue was embedded in OCT embedding medium (Sakura Finetek USA) and cut into 30 and 15 μm thick sections using a cryostat (RWD, China). Frozen sections were mounted on polylysine-coated slides and air-dried at 37°C, followed by staining or storage at −80°C. Hippocampal tissue for biochemical analysis was flash-frozen in liquid nitrogen immediately after microdissection and stored at −80°C until subsequent experiments.

### Western blotting

Hippocampal tissues were supplemented with a mixture of protein phosphatase inhibitor and PMSF in ice-cold RIPA buffer (R0010, Solarbio). Homogenized tissues were centrifuged at 13200 × *g* for 15 min, and the supernatant was quantified using a BCA protein quantification kit (CWBIO, CW0014S). An equal volume of total protein was loaded onto a 10% Bis-Tris SurePAGE gel for separation and then transferred to a 0.45 μm PVDF membrane. The membrane was blocked with 5% skim milk at room temperature for at least 2 h, incubated overnight with primary antibody at 4°C, and finally incubated for 2 h at room temperature with horseradish peroxidase (HRP)-labeled goat anti-rabbit IgG (ZB-2305, 1:5000, Zhongshan Jinqiao) and HRP-labeled goat anti-mouse IgG (ZB-2301, 1:5000, Zhongshan Jinqiao) secondary antibodies. Protein band signals were detected using ECL reagent, and quantification was performed using ImageJ software. The following primary antibodies were used: anti-PSD95 (Servicebio, GB11277, 1:1000), anti-NR2B antibody (Abcam, AB254356, 1:1000), anti-NR2A antibody (Abcam, AB124913, 1:1000), anti-GluA1 (Cell Signaling Technology, 13 185s, 1:1000), anti-GluA2 (Cell Signaling Technology, 13 607s, 1:1000), anti-synaptophysin (Cell Signaling Technology, 36 406s, 1:1000), anti-amyloid-β (Proteintech, 25 524-1-AP, 1:1000), anti-BDNF (Abcam, AB108319, 1:1000), anti-APP (Cell Signaling Technology, 2452S, 1:1000), anti-PS1 (Abcam, AB76083, 1:1000), anti-PKA (Abcam, AB32514, 1:1000), anti- pPKA (Cell Signaling Technology, 5661 s, 1:1000), anti- pCREB (Abcam, AB32096, 1:1000), anti-CREB (Abcam, AB32515, 1:1000), anti-PDE4A (ABclonal, A20162, 1:1000), anti-Bax (Servicebio, GB114122, 1:1000), anti-Bcl2 (Servicebio, GB154380, 1:1000), anti-TauSer214 (Abcam, AB170892, 1:1000), anti-TauSer404 (Abcam, AB92676, 1:1000), anti-Tau (Abcam, 66 499-1-IG, 1:1000). GAPDH or β-actin was used as a loading control to normalize the protein levels in each group.

### Immunofluorescence histochemistry

Brain sections (30 μm) were removed from −20°C, allowed to return to room temperature for 30 min, and washed with 1xPBST for 15 min. They were then incubated with 5% donkey serum (Servicebio, G1217) for 1 h. The sections were then incubated overnight at 4°C with anti-Aβ antibody (Proteintech, 25 524-1-AP, 1:200) and NeuN (Servicebio, GB11138, 1:1000). The next day, they were incubated with Cy3-labeled goat anti-rabbit IgG (Servicebio, GB21303, 1:400) at room temperature for 2 h, stained with DAPI for 10 min, washed three times with 1xPBST, and mounted with anti-fluorescence quenching mounting medium (Servicebio, G1401). Finally, images were acquired using a Leica confocal microscope, and statistical analysis was performed using ImageJ software.

### Nissl staining

Brain sections were removed from −20°C and allowed to warm to room temperature for 10 min, then immersed in pure water for 5 min before use. Nissl staining was performed using a kit (Servicebio, G1086). The sections were immersed in solution A for 15 min, rinsed twice with pure water, and then immersed in solution B for differentiation staining. The sections were then rapidly dehydrated with anhydrous ethanol (Shanghai Test, 10 009 265) for 5 s. Microscopic observation was performed until the Nissl bodies were clear and the background was nearly colorless. Finally, the sections were dried in a 65°C oven and mounted with neutral resin.

### Golgi staining

Freshly dissected brain tissue was stained using the FD Rapid Golgi Staining Kit (Servicebio, G1152). In short, the tissue was sequentially immersed in solutions A and B and incubated at room temperature for 2 weeks, then transferred to solution C and incubated at 4°C in the dark for 72 h. Subsequently, 150 μm thick fresh brain sections were prepared using a vibratory microtome (Leica VT 1200S), and staining was performed according to the manufacturer’s manual. Finally, dendritic spine images were acquired using a 60× objective lens, and the density, length, and dendritic complexity of dendritic spines in the CA1 region of the hippocampus were quantified using Fiji Image J software and the Sholl plugin.^33^

### Statistical analysis

All data are expressed as means ± SEM. Normality was verified using the Shapiro–Wilk test, and homogeneity of variance was verified using the Brown-Forsythe test. Differences among multiple groups were assessed for significance using two-way (genotype × treatment) ANOVA, followed by a Tukey post-hoc test. Graph plotting and statistical analysis were performed using GraphPad Prism 10.1.2 software. *P*-value < .05 was considered statistically significant.

## RESULTS

### Alcohol-induced synaptic damage in the hippocampus of 3xTg-AD mice was related to increased PDE4A expression

**Figure 1:**
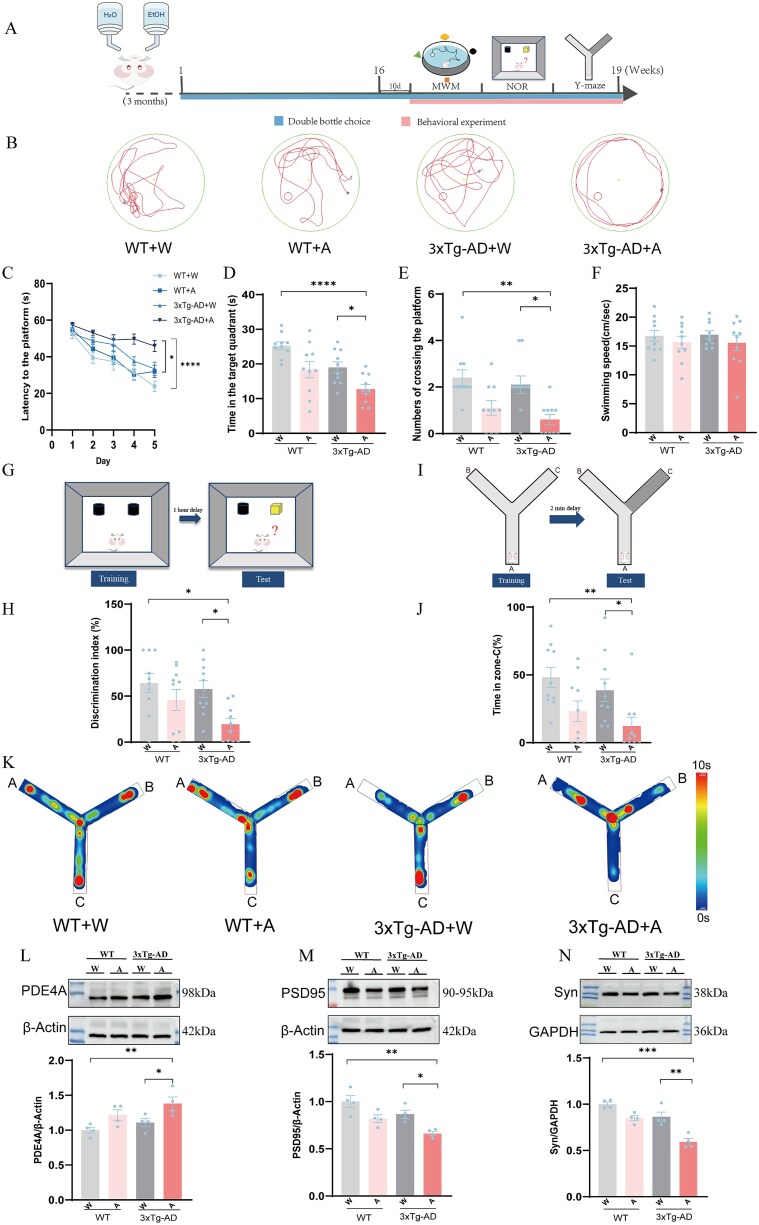
Alcohol exposure exacerbates the impairment of memory and synaptic proteins and upregulates hippocampal PDE4A expression in 3xTg-AD mice. (A) Experimental timeline: 3-month-old mice underwent a 19-week two-bottle free-choice paradigm with 25% (v/v) ethanol. Behavioral tests, including Morris water maze (MWM), novel object recognition (NOR), and Y maze, were conducted at week 17, n = 10 per group. (B) Representative swimming paths of mice on probe trial (day 6) of the MWM. (C) Escape latency across 5 consecutive training days in the MWM. (D) Time spent in the target quadrant on day 6. (E) Number of platform crossings on day 6. (F) Swimming speed of mice in each group. (G and H) Flow chart of NOR experiment and discrimination index. (I–K) Y maze flow chart, proportion of time spent in novel area C, and trajectory heat map. (L–N) Western blotting analysis of hippocampal PDE4A, PSD95, and synaptophysin protein levels, and quantitative analysis of protein expression levels. β-Actin/GAPDH served as the loading control. n = 4 per group. ^*^P < .05, ^**^P < .01, ^***^P < .001 compared with the 3xTg-AD + A group at the same time point.

To establish the AlD model, 3-month-old 3xTg-AD and WT mice were subjected to a two-bottle free-choice ethanol drinking paradigm (25% ethanol concentration) for 19 weeks (Figure 1A). Cognitive function was systematically assessed using a battery of behavioral tests, including the MWM, NOR, and Y-maze.

In the MWM acquisition phase (days 1-5), all groups exhibited a progressive reduction in escape latency over training days (Figure 1C), indicating intact baseline spatial learning. However, on day 5, the 3xTg-AD + A group displayed significantly prolonged escape latency compared to both the WT + W group (*P* < .0001) and the 3xTg-AD + W group (*P* < .05). During the spatial probe trial on day 6, the 3xTg-AD + A group showed pronounced spatial memory deficits, as evidenced by a significant decrease in the number of platform crossings (*P* < .05) and reduced time spent in the target quadrant (*P* < .05), with no differences in swimming speed among groups (Figure 1D–F).

In the NOR test, the novel object discrimination index was significantly lower in the 3xTg-AD + A group than in the WT + W (*P* < .05) and 3xTg-AD + W (*P* < .05) groups, indicating impairment in non-spatial memory (Figure 1G and H). The Y-maze test further revealed a significant reduction in novel arm exploration time in the 3xTg-AD + A group compared to WT + W (*P* < .01) and 3xTg-AD + W (*P* < .05) groups, suggesting compromised working memory (Figure 1I–K). Collectively, these behavioral findings demonstrate that chronic ethanol exposure exacerbates learning and memory deficits in 3xTg-AD mice.

Western blotting analysis of hippocampal tissues revealed a significant upregulation of PDE4A protein expression in the 3xTg-AD + A group compared to the 3xTg-AD + W group (*P* < .05), whereas no significant change was observed in the WT + A group (Figure 1L). Concurrently, the expression levels of key postsynaptic markers, PSD95 (*P* < .05) and synaptophysin (*P* < .01), were significantly reduced in the 3xTg-AD + A group relative to the 3xTg-AD + W group, with a similar downward trend noted in the WT + A group (Figure 1M and N).

These molecular alterations align closely with the observed behavioral impairments, suggesting that ethanol-aggravated cognitive dysfunction in 3xTg-AD mice may be linked to upregulated PDE4A expression and downregulated synaptic protein levels.

### PDE4A knockdown reversed alcohol-induced impairment of learning and memory in 3xTg-AD mice

**Figure 2:**
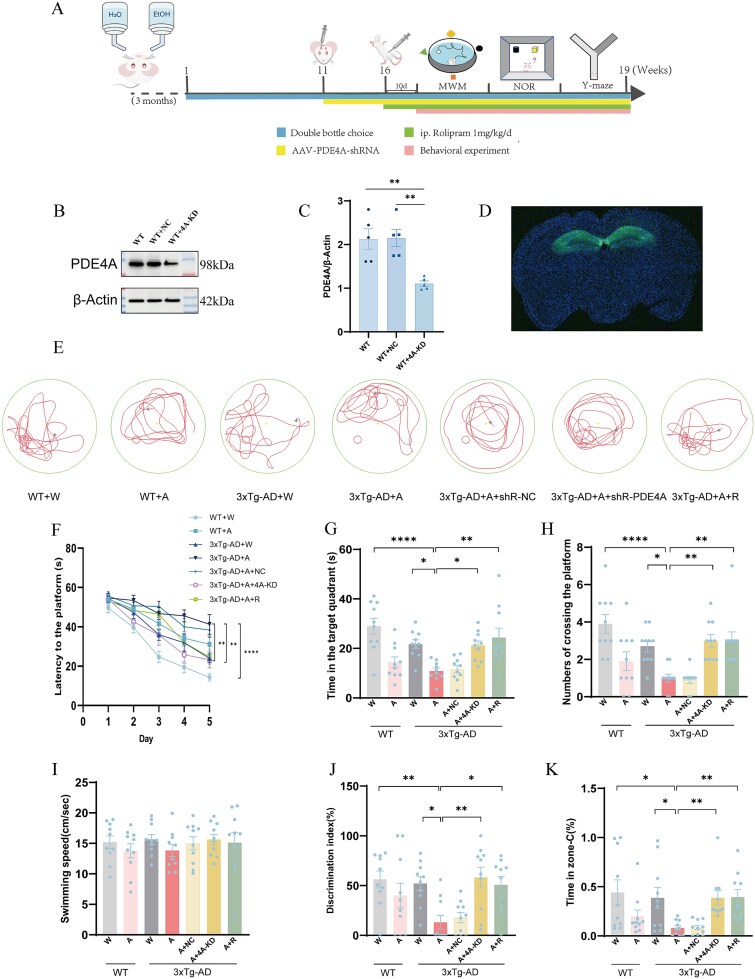
PDE4A knockdown reverses memory deficits in the Morris water maze (MWM), Y maze, and novel object recognition (NOR) tests in AlD mice. (A) Experimental timeline: 3-month-old mice underwent a 19-week two-bottle free-choice paradigm with 25% (v/v) ethanol. PDE4A knockdown at week 11 or rolipram injections at week 16. Behavioral tests, including MWM, NOR, and Y maze, were conducted at week 17. (B–D) Validation of PDE4A knockdown in the hippocampus. *n* = 5 per group. (E) Representative swimming paths of mice on probe trial (day 6) of the MWM. (F) Escape latency across 5 consecutive training days in the MWM. (G) Time spent in the target quadrant on day 6. (H) Number of platform crossings on day 6. (I) Swimming speed of mice in each group. (J) Discrimination index in the NOR test. (K) Proportion of time spent in the novel area C of the Y maze. Bars represent mean ± standard error of the mean (SEM). *n* = 10 per group, ^*^P < .05, ^**^P < .01, ^***^P < .001, ^****^P < .0001 compared with the 3xTg-AD + a group at the same time point.

To evaluate the therapeutic effect of PDE4A suppression, 3xTg-AD mice received bilateral hippocampal injections of AAV-PDE4A-shRNA (1 μL, 1.7 × 10^12^ vg/mL) in the 11th week to achieve targeted PDE4A knockdown. Beginning at week 16, a separate group received daily intraperitoneal injections of the PDE4 inhibitor rolipram for 21 days (Figure 2A). Western blotting analysis confirmed successful PDE4A knockdown, with protein expression reduced by ~50% compared to the control group (*P* < .01; Figure 2B–D).

In the MWM test, all groups exhibited a progressive decrease in escape latency over the training period. After confirming no intergroup differences in swimming speed (Figure 2I), we found that the 3xTg-AD + A group displayed significantly longer escape latency than the 3xTg-AD + W (*P* < .01) and WT + W (*P* < .0001) groups. Notably, both PDE4A-knockdown (3xTg-AD + A + 4A-KD) and rolipram-treated (3xTg-AD + A + R) groups showed markedly shortened escape latencies relative to the 3xTg-AD + A group (*P* < .01; Figure 2F).

Spatial memory retention was also significantly improved in PDE4A-knockdown mice, as evidenced by increased time spent in the target quadrant (*P* < .05) and a greater number of platform crossings (*P* < .01) compared to the 3xTg-AD + A group (Figure 2G and H). In the NOR test, the discrimination index was significantly higher in the 3xTg-AD + A + 4A-KD group than in the 3xTg-AD + A group (*P* < .01; Figure 2J), indicating rescued recognition memory. Similarly, in the Y-maze test, PDE4A-knockdown mice spent significantly more time exploring the novel arm than the 3xTg-AD + A group (*P* < .01; Figure 2K).

Together, these results demonstrate that targeted PDE4A knockdown effectively reverses alcohol-induced deficits in hippocampal-dependent memory and cognitive flexibility in 3xTg-AD mice.

### PDE4A knockdown attenuated Aβ pathology and Tau hyperphosphorylation in AlD mice

**Figure 3:**
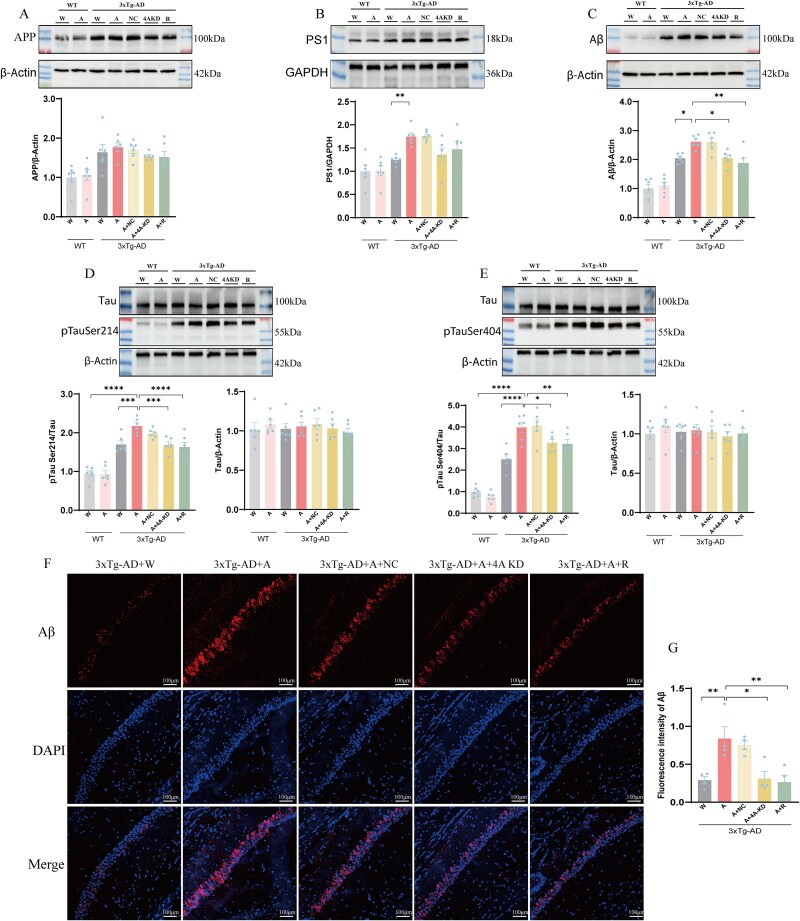
PDE4A knockdown alleviates Aβ pathology and tau hyperphosphorylation in the hippocampus of AlD mice. (A–E) Western blotting analysis of hippocampal APP, PS1, Aβ, pTauSer214, and pTauSer404 protein levels, and quantitative analysis of protein expression levels. β-Actin/GAPDH served as the loading control. *n* = 6 per group. (F-G) immunofluorescence staining and quantitative analysis of Aβ in the CA1 region of the hippocampus in mice, *n* = 4 per group. Bars represent mean ± SEM, ^*^P < .05, ^**^P < .01, ^***^P < .001, ^****^P < .0001 compared with the 3xTg-AD + A group at the same time point.

To investigate the effect of PDE4A knockdown on Aβ pathogenesis, we analyzed the expression of key proteins in the amyloidogenic pathway by Western blotting. Hippocampal levels of PS1 (*P* < .01) and Aβ (*P* < .05) were significantly upregulated in the 3xTg-AD + A group compared with the 3xTg-AD + W group. PDE4A knockdown significantly suppressed the alcohol-induced increase in Aβ levels (*P* < .05; Figure 3B and C), although APP expression remained unchanged across groups (Figure 3A).

We next examined Tau phosphorylation at residues Ser214 and Ser404. The 3xTg-AD + A group showed markedly elevated phosphorylation levels at both Tau Ser214 (*P* < .001) and Ser404 (*P* < .0001) compared to the 3xTg-AD + W controls. Notably, PDE4A knockdown significantly reduced phosphorylation at Ser214 (*P* < .001) and Ser404 (*P* < .05) in AlD model mice (Figure 3D and E).

Consistent with these biochemical findings, immunofluorescence results further revealed significantly increased Aβ deposition in the hippocampal CA1 region of the 3xTg-AD + A group relative to the 3xTg-AD + W group (*P* < .01). This pathological deposition was markedly attenuated following PDE4A knockdown (*P* < .05; Figure 3F and G).

### PDE4A knockdown rescued synaptic structural integrity and glutamate receptor expression in AlD mice

**Figure 4:**
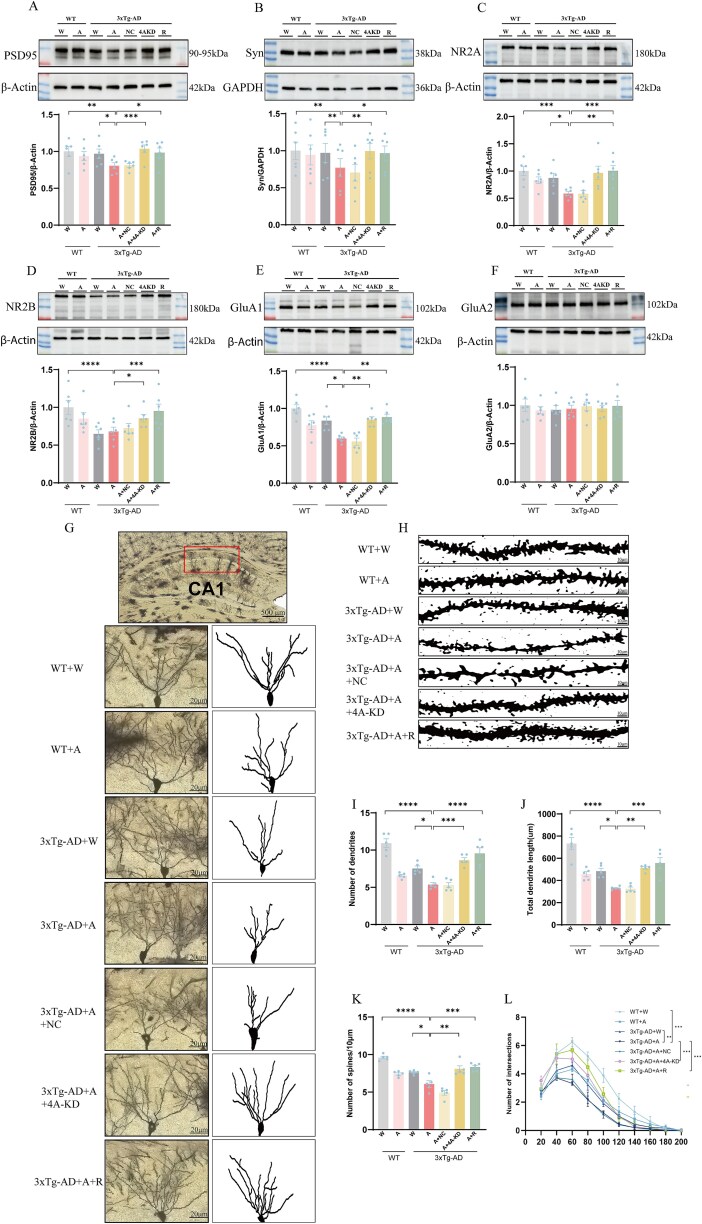
PDE4A knockdown rescues synaptic morphological and structural impairments in AlD mice. (A–F) western blotting analysis and quantitation of hippocampal protein levels of PSD95, Syn, NR2A, NR2B, GluA1, and GluA2. β-Actin/GAPDH served as the loading control. *n* = 6 per group. (G and H) The morphology and density of dendritic spines in the CA1 region of the hippocampus were observed using Golgi staining. Number of dendritic spine branches (I), total length (J), and density (K). Sholl analysis of synaptic dendritic spine complexity (L). *n* = 5 per group. Data are expressed as mean ± SEM. Statistical significance: ^*^P < .05, ^**^P < .01, ^***^P < .001, ^****^P < .0001 vs. 3xTg-AD + A group in the same period.

To investigate the role of PDE4A in synaptic molecular homeostasis, we assessed the expression levels of synaptic markers (PSD95, Synaptophysin) and glutamate receptor subunits (NR2A, NR2B, GluA1, GluA2) in hippocampal tissue by Western blotting. Compared with the 3xTg-AD + W group, the 3xTg-AD + A group exhibited significant downregulation of the postsynaptic protein PSD95 (*P* < .05) and the presynaptic protein Synaptophysin (*P* < .01). These reductions were effectively reversed in the 3xTg-AD + A + 4A KD group, with PSD95 (*P* < .001) and Synaptophysin (*P* < .01) levels significantly restored (Figure 4A and B).

Furthermore, the expression of the NMDA receptor subunit NR2A (*P* < .05) and the AMPA receptor subunit GluA1 (*P* < .05) was significantly lower in the AlD model group than in the 3xTg-AD + W controls. PDE4A knockdown markedly increased the expression of both NR2A (*P* < .01) and GluA1 (*P* < .01; Figure 4C and E). In contrast, NR2B expression did not differ significantly between the 3xTg-AD + A and 3xTg-AD + W groups, though both were lower than the WT + W group (*P* < .0001; Figure 4D). PDE4A intervention significantly restored NR2B expression (*P* < .05). Notably, GluA2 levels remained unaltered across all 3xTg-AD groups, suggesting its regulation is independent of the PDE4A pathway (Figure 4F).

We next employed Golgi staining to evaluate neuronal morphological integrity (Figure 4G and H). The 3xTg-AD + A group showed significant reductions in dendritic branch number, total dendritic length, and spine density compared to the 3xTg-AD + W group (*P* < .05 for all). In contrast, PDE4A knockdown markedly improved these structural parameters, increasing branch number (*P* < .001), total dendritic length (*P* < .01), and spine density (*P* < .01; Figure 4I–K), indicating a restorative effect on synaptic architecture.

Sholl analysis further revealed that dendritic complexity was significantly impaired in the 3xTg-AD + A group compared to both WT + W (*P* < .001) and 3xTg-AD + W (*P* < .01) groups. PDE4A knockdown substantially enhanced the Sholl profile (*P* < .001), with a particularly prominent increase in dendritic intersections within the 40–80 μm radial range (Figure 4L), underscoring the role of PDE4A in maintaining dendritic arborization.

### PDE4A knockdown attenuated neuronal damage and apoptosis in AlD mice

**Figure 5:**
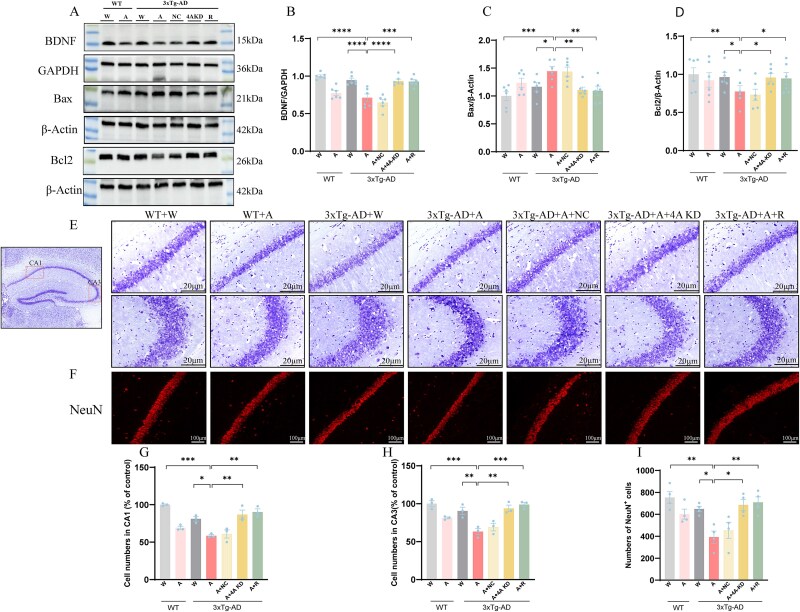
PDE4A knockdown attenuates neuronal damage and apoptosis in AlD mice. (A–D) Western blotting analysis of hippocampal BDNF, Bax, and Bcl2 protein levels, and quantitative analysis of protein expression levels. β-Actin/GAPDH served as the loading control. *n* = 6 per group. (E) Results of Nissl staining of the hippocampus in each group of mice. Quantitative statistics of Nissl bodies in hippocampal CA1 (G) and CA3 (H); *n* = 3 per group. Immunofluorescence staining and quantitative analysis of NeuN^+^ in the CA1 region of the hippocampus in mice (F, I); *n* = 4 per group. Bars represent mean ± SEM. ^*^P < .05, ^**^P < .01, ^***^P < .001, ^****^P < .0001 compared with the 3xTg-AD + A group at the same time point.

To assess neuronal integrity under alcohol exposure, we measured the expression of BDNF and key apoptotic regulators in the hippocampus by western blotting (Figure 4A). The 3xTg-AD + A group exhibited a significant decrease in BDNF levels compared to the 3xTg-AD + W group (*P* < .0001). PDE4A knockdown effectively restored BDNF expression in AlD model mice (*P* < .0001; Figure 4B). Furthermore, alcohol-exposed mice showed marked upregulation of the pro-apoptotic protein Bax (*P* < .05) and downregulation of the anti-apoptotic protein Bcl2 (*P* < .05) relative to the 3xTg-AD + W group. PDE4A knockdown significantly reversed these alterations, reducing Bax (*P* < .01) and elevating Bcl2 (*P* < .05) expression (Figure 5C and D).

To further verify the role of the neuroprotective effect of PDE4A suppression, we performed Nissl staining on hippocampal sections. The number of Nissl bodies was significantly reduced in the CA1 (*P* < .05) and CA3 (*P* < .01) regions of the 3xTg-AD + A group compared to the 3xTg-AD + W group. PDE4A knockdown markedly increased Nissl bodies count in both CA1 (*P* < .01) and CA3 (*P* < .01) regions (Figure 5G and H), indicating restored neuronal metabolic activity.

Consistent with these findings, immunofluorescence analysis revealed a significant decrease in NeuN^+^ cells in the 3xTg-AD + A group compared to the 3xTg-AD + W group (*P* < .05). This loss of neurons was effectively rescued by PDE4A knockdown, which significantly increased the number of NeuN^+^ cells compared to the AlD model group (*P* < .05; Figure 5I).

### PDE4A knockdown upregulated the expression of key proteins in the PKA/CREB pathway

**Figure 6:**
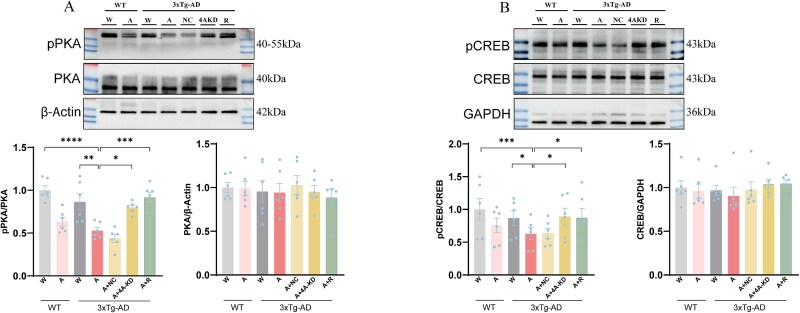
PDE4A knockdown upregulates the expression of key proteins in the PKA/CREB pathway in AlD mice. (A and B) Western blotting analysis of hippocampal PKA and CREB protein levels, and quantitative analysis of protein expression levels. β-Actin/GAPDH served as the loading control. *n* = 6 per group. Bars represent mean ± SEM. ^*^P < .05, ^**^P < .01, ^***^P < .001, ^****^P < .0001 compared with the 3xTg-AD + A group at the same time point.

To investigate the key molecular changes in the cAMP/PKA/CREB signaling pathway in hippocampal tissue, Western blotting analysis was performed to quantitatively analyze the protein expression levels of PKA catalytic subunit and phosphorylated CREB (Ser133 site) in hippocampal tissues. Compared with the WT + W group, the 3xTg-AD + W group had already shown decreased expression levels of p-PKA and p-CREB. This reduction was further exacerbated by long-term alcohol exposure in the 3xTg-AD + A group for p-PKA (*P* < .01) and p-CREB (*P* < .05; Figure 6A and B). Notably, PDE4A knockdown intervention significantly reversed the downregulation of p-PKA (*P* < .05) and p-CREB (*P* < .05).

### PDE4A knockdown reduced alcohol consumption in AlD mice

**Figure 7:**
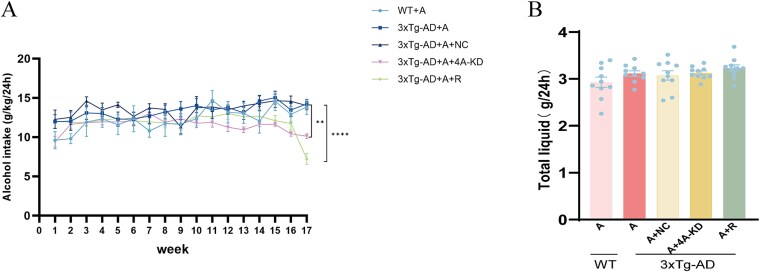
PDE4A knockdown reduces alcohol consumption in AlD model mice. (A) Trends in alcohol intake over weeks 1-17. (B) Total fluid intake at week 17. *n* = 10 per group. Data are expressed as mean ± SEM. Statistical significance: ^**^P < .01, ^****^P < .0001 vs. 3xTg-AD + A group during the same period.

To monitor the dynamics of alcohol consumption in mice, we recorded alcohol consumption in each group from weeks 1 to 19. Compared with the 3xTg-AD + A group, both the 3xTg-AD + A + 4A KD and 3xTg-AD + A + R groups showed significant reductions in alcohol consumption at week 17 (*P* < .01 and *P* < .0001, respectively; Figure 7A). Notably, total daily fluid intake did not change significantly in either group during the 17-week experimental period (Figure 7B).

## DISCUSSION

This study established a key role for PDE4A in the pathogenesis of AlD and delineated its underlying molecular mechanism. We demonstrated that selective PDE4A knockdown significantly ameliorated cognitive deficits in alcohol-exposed 3xTg-AD mouse models. The neuroprotective effects were primarily mediated through activation of cAMP/PKA/CREB signaling, along with attenuation of hippocampal neuronal apoptosis and synaptic injury.

Our previous research has demonstrated that chronic alcohol consumption specifically upregulates PDE4A expression.^30^ Furthermore, recent evidence indicates that alcohol induces neuronal apoptosis and synaptic damage, leading to cognitive impairment, suggesting a synergistic interaction between alcohol exposure and AD pathology.^9^^,^^34^^,^^35^ The hippocampus, a brain region central to learning, memory, and addictive behaviors, has become a key site for this dual impairment.^36–38^

PDE4A, a key isoform of the PDE4 family, is highly expressed in dementia-related regions such as the hippocampus and cerebral cortex, but shows minimal presence in the medullary vomiting center.^25^^,^^39–41^ This distribution suggests that targeting PDE4A may circumvent the emetic side effect commonly associated with broad-spectrum PDE4 inhibitors. Previous studies have linked PDE4A to cognitive and emotional regulation: mice with reduced PDE4A expression exhibited enhanced cognitive abilities^28^ and protection against MK-801-induced neurotoxicity.^42–44^ Moreover, elevated PDE4A5 impairs long-term memory and synaptic plasticity by inhibiting the cAMP-PKA signaling pathway.^45^^,^^46^ Therefore, given the synergistic effects of alcohol and AD pathology, we have focused on PDE4A as a potential therapeutic target.

Extensive studies indicate that chronic alcohol exposure promotes Aβ production and pathological Tau phosphorylation.^15^^,^^47^^,^^48^ These two pathological proteins further synergistically inhibit the transcriptional activity of CREB; leading to decreased expression of the downstream neurotrophic factor BDNF,^49^^,^^50^ thereby impairing neuronal survival.^51^^,^^52^ Notably, impaired neuronal function in turn exacerbates Aβ deposition and Tau pathology,^53^^,^^54^ ultimately forming a vicious cycle that accelerates disease progression. In this study, PDE4A knockdown significantly ameliorated cognitive impairment in AlD mice, an effect associated with increased hippocampal cAMP levels and phosphorylation activation of PKA/CREB pathway. Further mechanistic investigation revealed that PDE4A knockdown not only effectively alleviated core neurodegenerative pathological changes, such as Aβ deposition and abnormal Tau phosphorylation at Ser214/Ser404, but also significantly enhanced BDNF expression, collectively reducing neuronal loss and exerting robust neuroprotection.

At the synaptic level, presynaptic synaptophysin—a vesicle-associated protein—not only marks synaptic presence but also actively regulates neurotransmitter release and plasticity.^55^ Postsynaptic density protein 95 (PSD-95) serves as a scaffold that anchors and regulates NMDA and AMPA receptors, organizing postsynaptic signaling.^56^ Within the NMDA receptor, the NR2A subunit promotes synaptic strengthening and neuronal homeostasis via CREB pathway activation, whereas NR2B supports cognitive function under physiological conditions but may mediate excitotoxicity under pathological conditions.^57^ Our experimental results showed that, compared with the 3xTg-AD + W group, AlD model mice exhibited significant downregulation of hippocampal PSD-95, synaptophysin, and NR2A protein expression, whereas NR2B levels remained unchanged—possibly due to compensatory maintenance. PDE4A knockdown effectively restored the expression of these downregulated proteins. We also examined the two core subunits of AMPA receptors and found reduced GluA1 protein levels in AlD mice, which were normalized following PDE4A knockdown; GluA2 expression remained unaltered.

In the complex progression of AD, chronic alcohol exposure is recognized as a key driver of irreversible neuronal damage.^35^ Alcohol and its metabolites, such as acetaldehyde, can severely disrupt the dynamic balance of apoptosis regulatory networks within neurons.^58^ This imbalance, characterized by overactivation of pro-apoptotic signaling pathways (eg, upregulation of the pro-apoptotic protein Bax) and significant suppression of key anti-apoptotic mechanisms (eg, weakening the function of the protective protein Bcl2 and reducing neurotrophic factor support), disrupts neuronal homeostasis and drives cells toward apoptosis.^59–61^ Our studies demonstrate that PDE4A knockdown reestablishes this balance by downregulating Bax, upregulating Bcl2, and enhancing BDNF expression, thereby strengthening endogenous neuroprotection and attenuating alcohol-induced apoptosis.

## CONCLUSION

**Figure 8:**
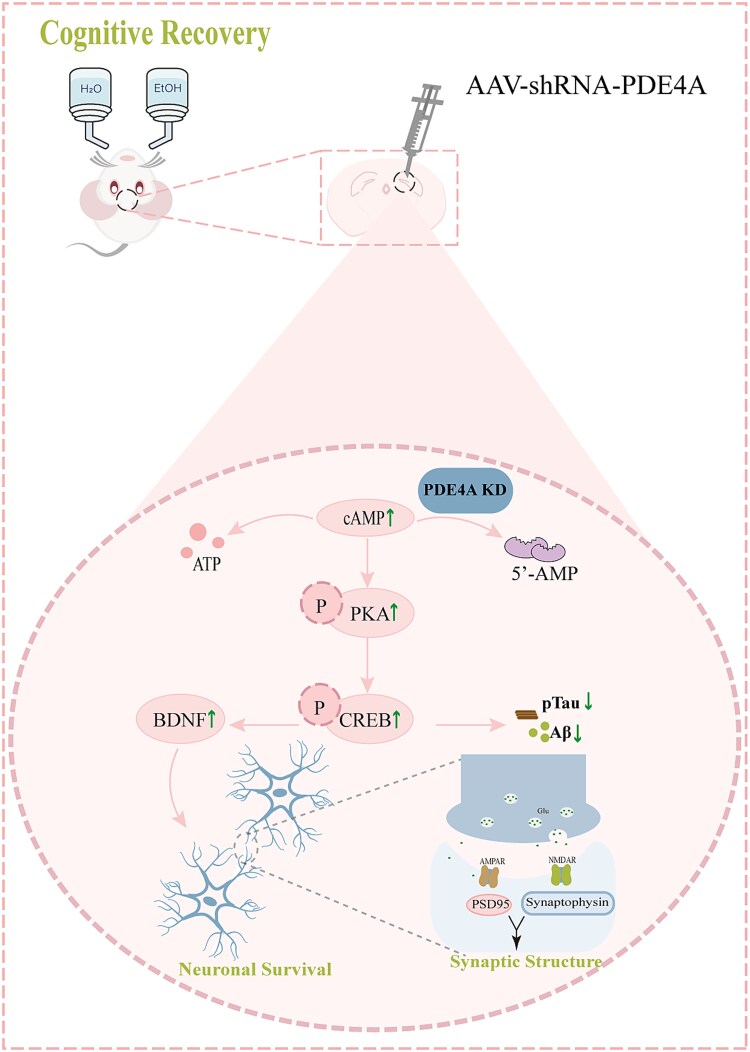
Schematic diagram of the intracellular signaling pathway underlying the therapeutic effect of PDE4A knockdown in AlD. Targeted knockdown of PDE4A elevates cerebral cyclic adenosine monophosphate (cAMP) levels by attenuating its hydrolysis. Increased cAMP enhances the activation of protein kinase A (PKA), which catalyzes the phosphorylation of the transcription factor CREB. Phosphorylated CREB in turn orchestrates two key neuroprotective responses: (1) transcriptional upregulation of brain-derived neurotrophic factor (BDNF), which stimulates the expression of synaptic markers (PSD95 and synaptophysin) and repairs synaptic damage; and (2) suppression of core Alzheimer-like pathologies, including Aβ accumulation and tau hyperphosphorylation. Concurrently, this signaling axis inhibits neuronal apoptosis. Together, these mechanisms underlie the observed amelioration of cognitive impairment in AlD.

In summary, PDE4A knockdown conferred neuroprotection and rescued cognitive function in AlD mice primarily by activating the cAMP/PKA/CREB signaling pathway (Figure 8). This activation underlay the observed mitigation of AD-like pathologies—including Aβ accumulation and Tau hyperphosphorylation—and the restoration of synaptic structure and function. The intervention further promoted neuronal survival by rebalancing apoptotic regulators (reduced Bax, increased Bcl-2). These results highlight PDE4A as a promising and precise therapeutic target for combating cognitive decline related to AlD.

## Data Availability

The original contributions are included in the article; further inquiries can be directed to the corresponding authors.
